# Inertial Sensor Angular Velocities Reflect Dynamic Knee Loading during Single Limb Loading in Individuals Following Anterior Cruciate Ligament Reconstruction

**DOI:** 10.3390/s18103460

**Published:** 2018-10-15

**Authors:** Kristamarie A. Pratt, Susan M. Sigward

**Affiliations:** 1 Human Performance Laboratory, Division of Biokinesiology and Physical Therapy, University of Southern California, 1540 E Alcazar St., CHP-155, Los Angeles, CA 90089-9006, USA; sigward@pt.usc.edu; 2 Department of Rehabilitation Sciences, University of Hartford, 200 Bloomfield Ave, West Hartford, CT 06117, USA

**Keywords:** inertial sensors, anterior cruciate ligament, rehabilitation, knee, gyroscope, power, angular velocity

## Abstract

Difficulty quantifying knee loading deficits clinically in individuals following anterior cruciate ligament reconstruction (ACLr) may underlie their persistence. Expense associated with quantifying knee moments (KMom) and power (KPow) with gold standard techniques precludes their use in the clinic. As segment and joint kinematics are used to calculate moments and power, it is possible that more accessible inertial sensor technology can be used to identify knee loading deficits. However, it is unknown if angular velocities measured with inertial sensors provide meaningful information regarding KMom/KPow during dynamic tasks post-ACLr. Twenty-one individuals 5.1 ± 1.5 months post-ACLr performed a single limb loading task, bilaterally. Data collected concurrently using a marker-based motion system and gyroscopes positioned lateral thighs/shanks. Intraclass correlation coefficients (ICC)(2,k) determined concurrent validity. To determine predictive ability of angular velocities for KMom/KPow, separate stepwise linear regressions performed using peak thigh, shank, and knee angular velocities extracted from gyroscopes. ICCs were greater than 0.947 (p < 0.001) for all variables. Thigh (r = 0.812 and r = 0.585; p < 0.001) and knee (r = 0.806 and r = 0.536; p < 0.001) angular velocities were strongly and moderately correlated to KPow and KMom, respectively. High ICCs indicated strong agreement between measurement systems. Thigh angular velocity (R^2^ = 0.66; p < 0.001) explained 66% of variance in KPow suggesting gyroscopes provide meaningful information regarding KPow. Less expensive inertial sensors may be helpful in identifying deficits clinically.

## 1. Introduction

Marker-based three-dimensional motion analysis is the current gold standard for quantification of movement deficits during dynamic tasks following knee injury or surgery. This technology uses three-dimensional marker positions (recorded at 250–340 Hz), and ground reaction force data (1360–1500 Hz) to calculate knee moments, angular velocities, and power. However, these analyses are complex, expensive, and time-consuming; thus, impractical in clinical settings. Two-dimensional video assessment using traditional video cameras or tablets (recording at 24–32 Hz) are becoming popular among clinicians for detection of movement impairments, but are limited to quantification of kinematics during tasks performed at slower speeds. Unfortunately, relevant knee loading deficits often coincide with much smaller differences in joint angle often making them difficult to observe clinically [1,2]. Differences in angles may be particularly difficult to detect during more dynamic tasks as individuals go through nearly 30–50 degrees of flexion in less than 200 milliseconds. Recent advances in wireless capabilities and data storage in wearable technology make inertial sensors more affordable and practical for movement assessments outside a motion analysis laboratory [3,4,5,6,7].

Individuals following anterior cruciate ligament (ACL) reconstructive surgery present with altered sagittal plane knee loading patterns that persist 6 to 24 months post-surgery. This coincides with the time when they are performing more demanding functional tasks and returning to participation in higher levels of physical activities and sports [8,9,10,11]. The presence of altered loading at this time is of particular concern as it is related to an increased risk for re-injury. A recent prospective study found that the odds of suffering a second ACL injury were 3.3 times greater in those who exhibited asymmetrical knee loading during a drop land at the time they returned to sports [12]. Biomechanically altered sagittal plane knee loading patterns following ACL reconstruction (ACLr) are characterized by decreased knee power absorption, angular velocities, and extensor moments in the reconstructed knee when compared to non­surgical knee and healthy controls. They are commonly observed during portions of dynamic tasks that require eccentric control or deceleration (e.g., running, landing, hopping) [2,11,13,14]. Clinicians often rely on measures of function (i.e., how far an individual can hop) to assess knee function [15,16]; however, these assessments are not sensitive to altered knee mechanics identified with gold standard motion capture technology [1]. Marker-based three-dimensional motion analysis revealed that individuals who were able to pass clinical hop tests, determined by comparing the distance hopped between reconstructed and healthy limbs, continued to exhibit a 6% decrease in knee extensor moment and 43% decrease in knee power absorption in the reconstructed limb [1]. The inability to identify specific mechanical loading deficits with clinical assessments may underlie their persistence. Given the potential long-term consequences of persistent deficits, it is critical to develop clinically useful methods for identification and improvement of altered loading patterns beyond functional measures of performance.

Inertial sensors (accelerometer, gyroscope, and magnetometer) are commonly used to detect events or to calculate spatial-temporal variables; however, they can be affixed to body segments to measure segment angular velocities and accelerations. Despite the fact that kinematics are not the only variables considered in the calculation of power and joint moments, kinematic variables, specifically joint and segment angular velocities, may adequately reflect knee power absorption and knee extensor moments during dynamics tasks. Knee as well as thigh and shank angular velocities not only quantify how fast a segment rotates, but also provide a preliminary understanding of neuromuscular control of dynamic tasks. Individuals may employ subtle changes in joint kinematics and concurrently, segment kinematics, to alter forces and decrease knee loading during these dynamic tasks [17,18]. While calculations may be performed to quantify segment/joint angles using multiple sensors, recent studies have highlighted the ability of raw outputs of a single sensor to detect between limb differences in knee loading during gait [19,20] and a single limb loading task [17,21]. In individuals following ACLr between limb ratios in shank [19] and thigh [21] angular velocities have been related to knee moment and power deficits during gait and a single limb loading task, respectively. The strength of the relationship with knee power ratios during single limb loading resulted in strong diagnostic accuracy of between limb deficits in knee joint power using thigh angular velocity measured with inertial units [21].

These data suggest that inertial sensors may be able to provide a more accessible alternative to marker-based three-dimensional motion analysis for detection of loading impairments in a clinical setting, particularly during a single limb loading task. This is important as a recent study found that individuals progressing to running after ACLr exhibit deficits in not only knee power but also in knee angular velocity and knee extensor moments during this less demanding single limb loading task [22]. Quantification of these deficits outside of laboratory setting will allow rehabilitation specialist to ensure they are being addressed. This study aims to advance the previous work relating between limb ratios in inertial sensor outputs of segment angular velocity to knee power deficits during a single limb loading task [21]. Using the same subjects, the current manuscript’s purpose is threefold: (1) to establish concurrent validity for measures of segment and joint angular between inertial sensors and the gold standard motion analysis system velocities, (2) to provide detailed methods for identifying inertial sensor outputs specific to knee power during the single limb loading task, and (3) to investigate the predictive value of between thigh and shank angular velocities and sagittal plane knee power and knee extensor moments in healthy and impaired limbs of individuals status-post ACLR during this task.

## 2. Materials and Methods

### 2.1. Participants

Twenty-one individuals (12 females; 28.8 ± 11.2 years) who had primary unilateral ACLr (11 right) using a bone-patellar-tendon-bone autograft, allograft, or hamstring autograft approximately 5.1 ± 1.5 months prior to testing participated. All participants reported that they were recreationally active prior to their injury (evaluated using Cincinnati Sports Activity questionnaire) [23]. Recreational athlete was defined as Level I or II on the Cincinnati Sports Activity scale. At the time of participation, individuals were actively attending physical therapy and had initiated a running progression within 2 months of testing.

Individuals were excluded from the study if they: (1) were not cleared by physical therapist to perform the functional activities, (2) had prior ACL injury and knee surgery on the contralateral limb, (3) had concurrent pathology or morphology that could cause pain or discomfort during physical activity, and (4) had any physical, cognitive, or other condition that may impair the individual’s ability to perform the tasks proposed in this study. An a priori power analysis performed using pilot data from six individuals determined that 14 subjects would provide more than 80% power at the alpha level of 0.05.

### 2.2. Instrumentation

Kinematic data and ground reaction force data were collected using either a marker-based, 11-camera motion capturing system (Qualysis Inc., Gothenberg, Sweden) at a 250 Hz and force platforms at 1500Hz (Advanced Mechanical Technologies, Inc., Newton, MA, USA) or a 14-camera motion capturing system (BTS Bioengineering Corp., Milan, Italy) at 340 Hz and force platforms at 1360 Hz (BTS Bioengineering Corp., Milan, Italy). Two motion capture systems were used due to a transition to a new motion capture system during the study. Concurrently, inertial data was collected using four inertial sensors equipped with a tri­axial accelerometers, gyroscopes, and magnetometers (Opal, APDM Inc., Portland, OR, USA). The primary variable of interest from the inertial sensors, angular velocity, was measured using the gyroscope. While direct measurements from the accelerometer and magnetometer were not used for analysis in this study, they remained active throughout data collection to increase accuracy of gyroscope measurements using APDM’s proprietary algorithm. The range for the gyroscope in X­ and Y­axes was ±34.9 rad/s and Z­axis is ±26.8 rad/s. The gyroscope’s noise density in X­ and Y­axes was 0.81 mrad/s/√Hz and 2.2 mrad/s/√Hz for Z­axis. Inertial data was recorded at 128Hz using Motion Studio software (APDM Inc., Portland, OR, USA). Data was synchronized and wirelessly streamed from all four sensors directly to the computer using “Robust Synchronized Streaming” mode. Data was buffered on the sensors to prevent data loss in the case of wireless interruptions.

### 2.3. Procedures

Testing took place in the University of Southern California’s Human Performance Laboratory located at Completive Athletic Training Zone, Pasadena, CA, USA. All procedures were explained to each participant and informed consent was obtained as approved by the Investigational Review Board at University of Southern California Health Sciences Campus. Parental consent was obtained for all individuals under the age of 18 years. After consenting to participate, participant’s age, height, weight, tibia length, knee medical history, and physical activity prior to injury were recorded.

Prior to testing, participants were asked to warm­up on a stationary bike for five minutes. Reflective markers were placed on first and fifth metatarsals, distal end of second toes, medial and lateral malleoli, medial and lateral epicondyles of femurs, greater trochanters, posterior superior iliac spines, iliac crests, and L5­S1 junction. In addition, tracking clusters, reflective markers attached to rigid plates, were secured bilaterally on participants’ thighs, lower legs and heels of their shoes by the same examiner. After the static calibration trial all markers were removed, except tracking clusters, pelvis, and distal toe markers which remained on during testing.

**Figure 1:**
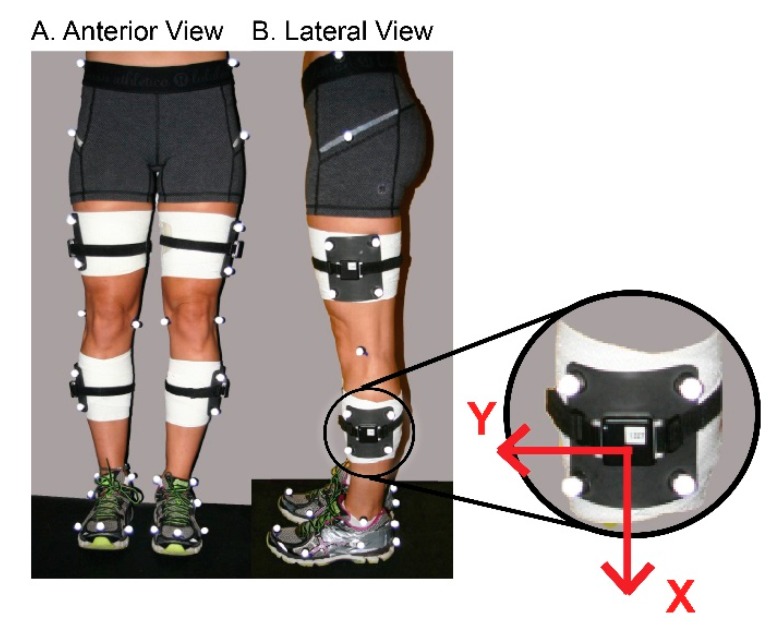
Orientation and location of inertial sensors and markers on thigh and shank during testing; Orientation of axes depicted on right.

Inertial sensors were placed on the mid-lateral thighs and shanks with the X­axis aligned superior–inferior, bilaterally. Care was taken to align the X­axis of thigh sensors with greater trochanters and lateral epicondyles of the femur, and X­axis of shank sensors with lateral epicondyles and lateral malleoli (Figure 1). For testing the position of inertial sensors coincided with the position of the tracking marker clusters; therefore, they were affixed to the rigid plates firmly using elastic straps and tape.

### 2.4. Single Limb Loading Test

**Figure 2:**
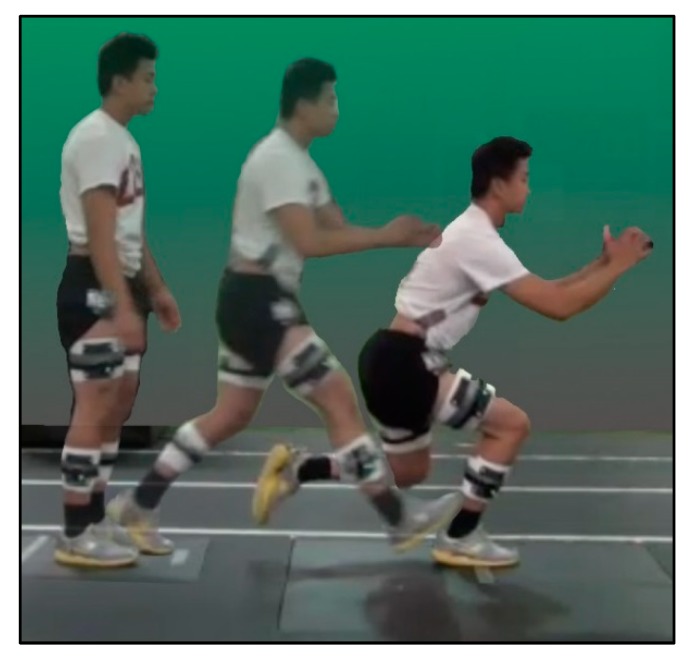
Single Limb Loading Test.

During testing, wearing their own athletic shoes, participants performed a dynamic single limb loading (SLL) task on each limb as described previously [21,22] (Figure 2). For this task, participants were instructed to stand on both feet on a single platform behind a tape line facing a target positioned on an adjacent force platform. The target was positioned a distance that was normalized to the length of each individual’s tibia. Participants were instructed to leap forward onto the target on a single limb, to lower as far they can and then return to the start on two limbs. Participants were asked to complete the task in one fluid movement without pausing. To encourage fluid and continuous movement they performed three consecutive repetitions at their own self-selected pace for each trial. Participants performed SLL trials alternating between limbs beginning with the non­surgical limb. A trial was considered acceptable when it contained presence of a distinct flight phase, maintenance of balance throughout the task and complete foot placement on the target force platform. The presence of a flight phase was considered as criteria for a successful trial to avoid instances of double limb support. It was determined by the absence of forces on either force plate prior to foot contact on the target force platform. Practice trials were allowed for individuals to become familiar with the task. Participants performed three trials on each limb.

### 2.5. Data Analysis

Reconstructed three-dimensional marker coordinates (Qualysis Inc. Tracking Manager, Gothenberg, Sweden or BTS Bioengineering Corp SMARTtracker, Milan, Italy) were used in combination with force platform and anthropometric data to calculate joint kinematics, kinetics and energetics (Visual3DTM, Version 4.8, C-Motion, Inc., Rockville, MD, USA). Coordinate data was filtered using a fourth order, low pass, zero-lag Butterworth filter with frequency cut-off of 12 Hz. Data from the standing calibration trial was used to derive the local coordinate systems of body segments. Lower extremity segments were modeled as a frusta of cones, while the pelvis was modeled as a cylinder. Six degrees of freedom of each segment were calculated by transforming the triad of markers to the position and orientation of each segment during the standing calibration trial. Euler angles were used to calculate joint kinematics in the subsequent order: flexion/extension, abduction/adduction, and internal/external rotation. Joint angles were expressed as movement of the distal segment relative to the proximal segment. Standard inverse dynamic equations used kinematics, anthropometrics, and ground reaction forces to calculate internal net joint moments. Net joint power was calculated as the product of joint moment and joint angular velocity and normalized to body mass. Segment angular velocities measured with the marker-based motion analysis system were calculated with respect to the global coordinate system. Data obtained from Visual3D^TM^ were exported and analyzed using a customized MATLAB^®^ program (Version R2014b, The MathWorks, Matick, MA, USA).

**Figure 3:**
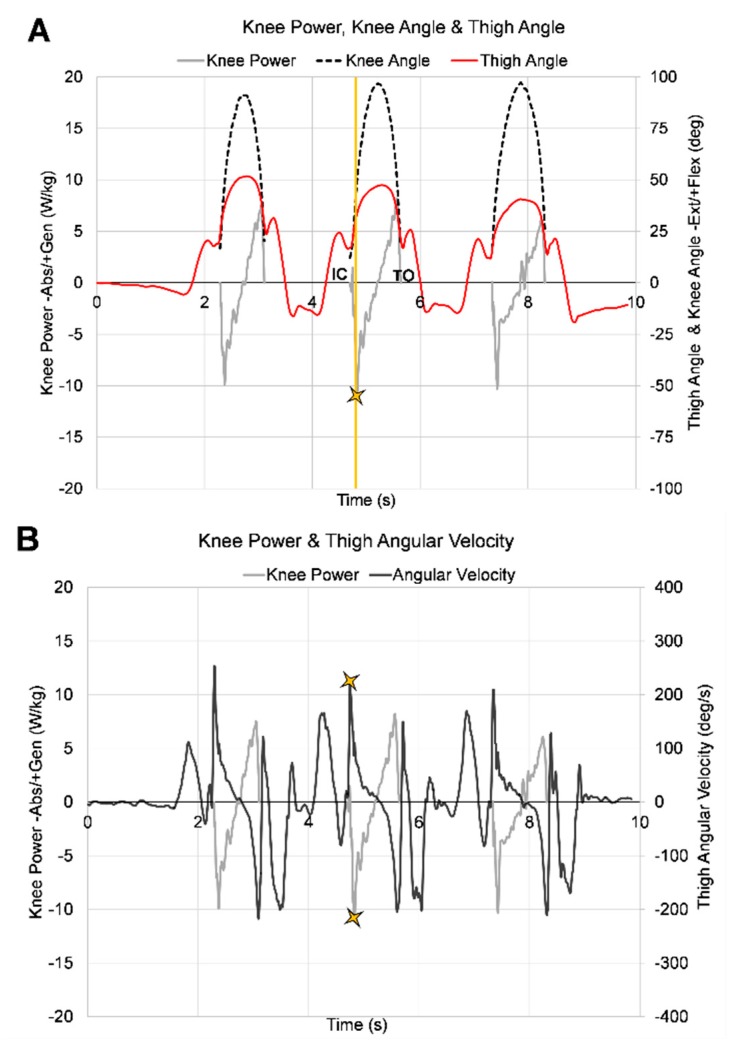
Three cycles of single limb loading task performed by one representative subject. (**A**) Marker-based knee angle (dashed black line), knee power (gray line), and inertial sensor thigh angle (red line); (**B**) Marker-based knee power (gray line) and inertial sensor thigh angular velocity (black line). Stars indicate the peak knee power absorption (**A**) and peak thigh angular velocity (**B**) identified after initial contact and before maximum knee flexion during the middle repetition of one trial.

Signals from the inertial sensors placed on thighs and shanks were used to measure thigh and shank angular velocity, respectively. Angular velocity, a direct output of the gyroscope, in the Z-plane of the sensor were chosen to represent sagittal plane movement (Figure 1). Thigh and shank angular velocity measurements were negated on the right limb to coincide with the global coordinate system where knee flexion involved positive rotation from vertical of the proximal thigh and negative rotation of the proximal shank segment from vertical. Segment angular velocity data was low-pass filtered using a fourth order zero-lag Butterworth filter with a 15 Hz cut­off frequency. Knee joint angular velocity was calculated as the sum of thigh and shank angular velocities at each time point throughout the movement with positive rotation representing knee flexion. Thigh angles in the sagittal plane were calculated as the integral of the thigh angular velocity with respect to time. A thigh segment angle of 0 degrees corresponds to a vertical position of the thigh with and an angle of 90 degrees to a horizontal position. Segment angles were used for the purposes of event identification in the inertial sensor data. For the single limb loading task, individuals began the task with the knee in more extension and the thigh segment more vertical. During execution of the task, individuals flexed their knee on a planted foot moving the thigh to a more horizontal position and then extended the knee returning the thigh to a more vertical position (Figure 3A)

All dependent variables were identified during the deceleration portion of stance phase of a single limb loading task. Stance phase was identified in the marker-based system using ground reaction forces, and in the inertial sensors using thigh angle measurements. For the marker-based motion capture system initial contact and toe-off were identified when the vertical ground reaction force was greater than 30 N and less than 30 N, respectively. For the inertial sensors, stance phase occurred between two local minimums of the thing angle, prior to and following a maximum thigh angle (Figure 3A, continuous red line). The local minimum thigh angle prior to the maximum thigh angle was initial contact and the local minimum thigh angle following was toe-off. Deceleration in the marker-based system was defined as the time between initial contact and peak knee flexion, and deceleration in the inertial sensors was defined as the time between the first local minimum thigh angle (initial contact) to the maximum thigh angle. Customized MATLAB^®^ programs were used to identify variables of interest extracted from inertial sensors.

During deceleration, peak knee power absorption (Figure 3A) and peak knee extensor moments were identified using the marker-based motion capture system. Peak knee, thigh (Figure 3B), and shank angular velocities in sagittal plane were identified using both the marker based system and inertial sensors during the deceleration phase. The average of three trials (middle repetition of each trial) of each limb (ACL reconstructed (ACLr), non­surgical (Non­Sx)) were used for analysis in both systems.

### 2.6. Statistical Analysis

To quantify the level of agreement between measurement systems, concurrent validity of shank, thigh, and knee angular velocity were determined using 2-way random intraclass correlation coefficients (ICC)(2,k). For clinical measurements agreement between measurement systems should exceed 0.90 to ensure reasonable validity.

To determine the best predictor of knee power absorption and knee extensor moment, two separate step-wise linear regressions were performed using shank, thigh, and knee angular velocities measured with inertial sensors. Peak knee power absorption (KPow) was the dependent variable for the first regression model and peak knee extensor moment (KMom) was the dependent variable for the second regression model. Peak shank (SAV), thigh (TAV), and knee (KAV) angular velocities from inertial sensors were independent variables. For both regression models, data from ACLr and Non-Sx limbs were considered together as initial multiple linear regression analysis that included limb as an independent variable determined that limb had no significant effect on the relationships (p = 0.072). Therefore, data presented below represents combined data from both limbs. One-tailed Pearson product–moment correlations were used to quantify the strength of the relationship between KPow and angular velocities and between KMom and angular velocities. A strong correlation was defined as a correlation greater than 0.75, a moderate correlation was defined as a correlation 0.50–0.75, and a weak correlation was defined as a correlation less than 0.5. Statistical analyses were performed using PASW software (version 18, SPSS, Inc., Chicago, IL, USA) with a significance level of α < 0.05.

## 3. Results

Descriptive statistics for 21 participants can be found in Table 1. High intraclass correlation coefficients (ICC > 0.90) indicated strong agreement between measurement systems for KAV, TAV, SAV during SLL (Table 2). 

**Figure 4:**
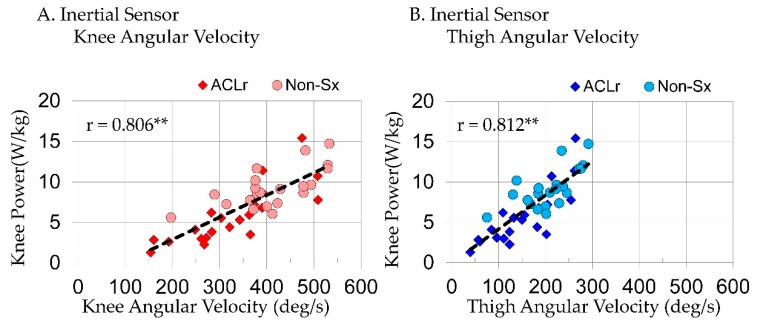
The relationship between peak knee power absorption and (**A**) peak knee angular velocities and (**B**) peak thigh angular velocities measured with inertial sensors in the reconstructed (ACLr) and nonsurgical (Non-Sx) limb; ** p < 0.001.

When considering joint and segment variables extracted from inertial sensors in a step-wise regression model predicting KPow, TAV(R^2^ = 0.660, p < 0.001) was the only variable to enter the model; it explained 66% of variance in KPow during SLL. Peak KAV(r = 0.806, p < 0.001; Figure 4A) and TAV(r = 0.812, p < 0.001; Figure 4B; Equation (1)) were strongly correlated and SAV(r = 0.596, p < 0.001) was moderately correlated with KPow.

Knee Power = 0.042(TAV) − 0.087(1)

**Figure 5:**
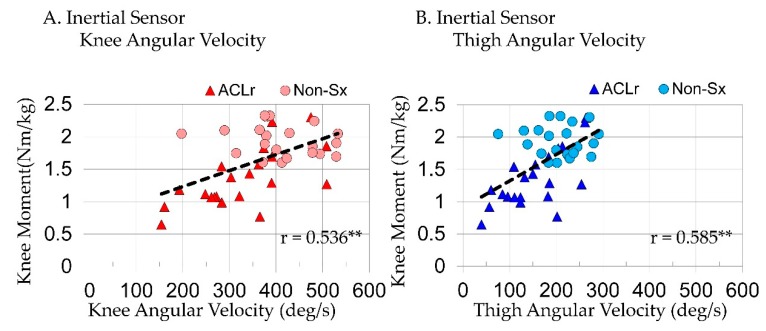
The relationship between peak knee extensor moments and (**A**) peak knee angular velocities and (**B**) peak thigh angular velocities measured with inertial sensors in the reconstructed (ACLr) and nonsurgical (Non-Sx) limb; ** p < 0.001.

TAV (R^2^ = 0.342, p < 0.001) was also the only variable to enter the KMom step-wise regression model when considering joint and segment variables extracted from inertial sensors. TAV explaining 34% of variance in KMom during SLL. Peak KAV(r = 0.536, p < 0.001; Figure 5A) and TAV(r = 0.585, p < 0.001; Figure 5B: Equation (2)) were moderately correlated with KMom and SAV(r = 0.345, p = 0.013) was poorly correlated.

Knee Moment = 0.004(TAV) + 0.585(2)

Joint and segment angular velocities were positively correlated with peak KPow and peak KMom indicating faster velocities were related to larger peak knee power absorption and peak knee extensor moments.

## 4. Discussion

Findings from this study provide a foundation for using inertial sensors to detect altered knee loading without presence of force plates or marker-based motion system during this SLL task. Previous studies demonstrated that individuals post-ACLr exhibit deficits in dynamic knee loading that are challenging to detect in the clinic [1,14,22,24]. The inability to quantify common knee loading deficits in the clinic in individuals following ACLr is concerning as post-surgery these individuals aim to return to high level dynamic tasks, where the knee plays an essential role in force attenuation [25,26,27].

The results of this study suggest that segment angular velocities measured with inertial sensors and marker-based motion analysis systems provide similar information supporting use of inertial sensors in the clinic. The agreement between the marker-based system and inertial sensors was high, with ICCs ranging between 0.94 and 0.989, when measuring knee and segment angular velocities. While inertial sensors are a direct measurement of segment angular velocities and marker-based measurements involve calculations from marker positions, strong intraclass correlation coefficients confirm that both methods may be used to quantify thigh and shank angular velocities during this task. In addition, knee angular velocities that involve calculations in both measurement systems, also had strong intraclass correlation coefficients. Together, these data confirm that direct measurements from the gyroscope of inertial measurement devices and calculated joint measurements provide a feasible alternative for marker-based motion analysis systems. Future work is needed to determine if the strength of this relationship is consistent across other functional tasks. 

Peak angular velocities coincided with peak knee power absorption just after initial contact (Figure 3B) as the knee is going into flexion (Figure 3A). When inertial sensor variables were considered together, sagittal plane peak thigh angular velocity was the best predictor of knee power absorption explaining 66% of the variance during single limb loading. After accounting for effects of thigh angular velocity, knee, and shank angular velocities did not add any additional information. The strength of the relationships suggests that angular velocity alone can provide meaningful information regarding knee power without marker-derived kinematics and ground reaction forces. This is not surprising as a between limb (surgical and non-surgical) ratio of thigh angular velocity in these same subjects was strong enough to identify knee power deficits with high sensitivity and specificity [21]. The correlation was slightly higher for thigh angular velocity; as a result, it was determined to be the stronger predictor of knee power when measured with inertial sensors.

Similarly, sagittal plane peak thigh angular velocity was the best predictor of peak knee extensor moments; however, it only explained 34% of the variance during single limb loading. Knee and shank angular velocities did not add any addition information. The moderate relationship between knee moment, and thigh (r = 0.585) and knee (r = 0.536) angular velocities suggests angular velocity measurements alone may provide some useful information regarding knee moment in the absence of ground reaction forces. However, it is not expected that these relationships are strong enough to add value to clinical decision making regarding knee moment deficits.

Using direct output of a single sensor on the thigh may be more practical for clinical use as it requires the purchase and application of fewer sensors. The use of a single sensor would provide clinicians with a simple tool that can be used to objectively quantify movement in the clinic and provide treatment rationales to third party payors. The strength of these relationships for knee power exceeded previously reported relationships between coronal plane thigh and shank angular velocities and knee adductor moments during single and double limb drop lands [18]. It is not surprising that segment angular velocity is more strongly related to joint power than moments given how they are calculated. Moreover, this SLL task required significant sagittal plane motion at the knee whereas frontal plane motion was limited. The testing procedures described in the current study allow for exploration of sagittal plane loading deficits commonly observed in individuals post-ACLr.

Shank angular velocity had a moderate relationship to knee power and poor relationship to knee moment. The range of angular velocities at the shank was smaller than those observed at the thigh and knee, but of similar magnitudes. When considered along with thigh angular velocity, shank did not add any additional information regarding knee power or moment. This suggests that motion at the thigh is more directly related to knee flexion during this single limb loading task. The instructions to perform the task encouraged individuals to lower themselves as far as they could. This may have resulted in large hip flexion angles increasing the contribution of thigh movement to knee flexion. However, future work is needed to determine if thigh and shank kinematics are reflective of hip and ankle kinematics, respectively.

Angular velocity measurements with inertial sensors provide meaningful information about an individual’s ability to accommodate forces through their knee following ACL reconstructive surgery during phases of tasks that are too quick for our eyes and traditional video recorders to capture. It is likely that thigh angular velocity measured with inertial sensors is highly sensitive to difference in power observed between limbs or changes over time as the regression equation indicated that a 0.042 deg/s change in thigh angular velocity coincides with a 1 W/kg change in knee power absorption (Equation (1)). Interestingly, limb did not influence the relationship between sagittal plane angular velocities and knee power or knee moment during this task despite the presence of between limb differences in angular velocities and knee power at this time point in rehabilitation. This supports the use of the non­surgical limb for comparison to assess knee loading asymmetries in the clinic, as seen commonly in assessment of rehabilitation progression [21,28,29,30]. While these findings set the foundation for quantifying knee power and moments with angular velocity measurements extracted from inertial sensors, they are limited to the single limb task assessed in this study. It is not clear if similar relationships exist during other dynamic tasks such running or hopping. For application of these data to the clinic, further work is needed to determine the sensitivity and specificity of these measures for quantifying altered knee loading.

## 5. Conclusions

Segment angular velocities measured with inertial sensor provide similar information to segment angular velocities measured with marker-based motion analysis systems. Furthermore, sagittal plane peak thigh angular velocity was the best predictor of peak knee power absorption and peak knee extensor moments. These findings suggest that in the absence of force platforms and a marker-based motion capture system, inertial sensors, more specifically a sensor placed on the thigh, may be useful in the clinic to quantify altered knee loading during a single limb loading task individuals following ACL reconstruction.

## Figures and Tables

**Table 1:** Descriptive statistics for the reconstructed (ACLr) and nonsurgical (Non-Sx) limb for joint and segment variables measured with marker-based motion capture and inertial sensor measurement systems; Data represents mean ± standard deviation and (range).

	ACLr Limb		Non-Sx Limb	
	Marker-Based	Inertial Sensor	Marker-Based	Inertial Sensor
Knee Power Absorption (W/kg)	5.8 ± 3.5 (1.3–15.4)	NA	9.2 ± 2.4 (5.6–14.7)	NA
Knee Extensor Moment (Nm/kg)	1.4 ± 0.4 (0.7–2.3)	NA	1.9 ± 0.2 (1.6–2.3)	NA
Knee Angular Velocity (deg/s)	328.0 ± 97.5 (154.4–521.2)	326.5 ± 100.6 (154.0–508.7)	420.1 ± 81.7 (249.6–543.3)	410.5 ± 84.55 (197.1–532.5)
Thigh Angular Velocity (deg/s)	156.0 ± 62.9 (48.7–276.0)	152.0 ± 67.2 (39.7–264.0)	210.0 ± 52.53 (108.0–308.0)	207.0 ± 54.0 (75.3–291.0)
Shank Angular Velocity (deg/s)	205.2 ± 53.8 (124.0–327.0)	195.1 ± 53.2 (119.8–301.3)	224.9 ± 33.79 (147.8–284.6)	221.5 ± 34.8 (125.7–270.7)

**Table 2:** Intraclass correlation coefficients (2,k) between marker-based motion capture and inertial sensor measurements for peak knee angular velocity, and peak thigh and shank angular velocities measured in all limbs, the reconstructed (ACLr) and nonsurgical (Non-Sx) limb.

	ALL Limbs	ACLr Limb	Non-Sx Limb
Knee Angular Velocity	0.978 **	0.989 **	0.950 **
Thigh Angular Velocity	0.967 **	0.947 **	0.973 **
Shank Angular Velocity	0.962 **	0.95 **	0.978 **

** Indicates significance; p < 0.001.
